# Insufficient preparedness of primary care practices for pandemic influenza and the effect of a preparedness plan in Japan: a prefecture-wide cross-sectional study

**DOI:** 10.1186/1471-2296-14-174

**Published:** 2013-11-19

**Authors:** Taro Tomizuka, Yasuhiro Kanatani, Kazuo Kawahara

**Affiliations:** 1 Department of Health Policy Science, Graduate School of Medical and Dental Science, Tokyo Medical and Dental University, 1-5-45 Yushima, Bunkyo-ku, Tokyo, Japan; 2 Department of Health Crisis Management, National Institute of Public Health, 2-3-6 Minami, Wako, Saitama, Japan

**Keywords:** Primary care practice, Primary care, Preparedness, Response, Pandemic preparedness plan, Personal protective equipment, Business continuity plan, Pandemic influenza

## Abstract

**Background:**

Cases of emerging infectious diseases, including H5N1 influenza, H7N9 influenza, and Middle East Respiratory Syndrome, have been reported in recent years, and the threat of pandemic outbreaks persists. In Japan, primary care is the frontline against emerging infectious diseases in communities. Although the importance of pandemic preparedness in primary care has been highlighted previously, few studies have thus far investigated the preparedness among primary care practices (PCPs) or differences in the preparedness of different institutional settings. We examined PCP preparedness and response to the 2009 influenza pandemic in Japan, and explored the role of a pandemic preparedness plan during the pandemic.

**Methods:**

We used a survey questionnaire to assess how well individual PCPs in Okinawa, Japan, were prepared for the 2009 influenza pandemic. The questionnaire was mailed to all eligible PCPs (N = 465) in Okinawa, regardless of their institutional setting. In addition, we assessed the differences in the preparedness of clinics and hospitals and determined whether the national preparedness plan affected individual preparedness and response. Data were analyzed using descriptive and logistic regression analyses.

**Results:**

A total of 174 (37.4%) PCPs responded to our survey. In general, high-level personal protective equipment (PPE) such as N95 masks (45.4%), gowns (30.5%), and eye protection (21.3%) was stocked at a low rate. Clinic-based PCPs were significantly less prepared than hospital-based PCPs to provide N95 masks (OR 0.34), gowns (OR 0.15), and eye protection (OR 0.18). In addition, only 32.8% of PCPs adopted an adequate business continuity plan (BCP). After controlling for institutional setting, reading the national preparedness plan was significantly associated with establishment of a BCP (OR 5.86), and with knowledge of how to transfer a swab specimen to a local medical laboratory (OR 5.60).

**Conclusions:**

With regard to PPE availability, PCPs (especially clinic-based PCPs) were not adequately prepared for the influenza pandemic. Awareness of the national pandemic preparedness plan is likely to promote prefecture-wide implementation of BCPs and surveillance activity.

## Background

Medical and public health authorities often warn the public about emerging respiratory infectious disease outbreaks. Two recent examples are avian influenza [1,2] and novel coronavirus infections such as Middle East Respiratory Syndrome [3,4]. In the past decade, cases of H5N1 and, more recently, H7N9 influenza infections, have been reported in Southeast and East Asian countries [5]. The fatality rates associated with these diseases are relatively high, but there appears to be limited human-to-human transmission. Nevertheless, the threat of a pandemic outbreak persists.

Primary care serves as the frontline against emerging infectious diseases. Primary care practices (PCPs) have an important role in treating and controlling the spread of these diseases in communities [6]. For example, a family physician in Canada examined, diagnosed, and treated the index case of the 2003 outbreak of Severe Acute Respiratory Syndrome (SARS) [7]. The index case in the 2009 H1N1 influenza pandemic in Japan was also examined by a family physician, in Kobe, Hyogo. The patient had no previous contact with infected foreigners, nor did he have a history of travel to areas of epidemic 2009 H1N1 influenza [8].

Because PCPs are likely to examine patients who present with acute respiratory illness, they are at risk of contracting emerging infectious diseases [9]. Therefore, it is possible for PCPs to have unexpected direct contact with infected patients [10]. Direct contact between PCPs and patients in health care settings is a major risk factor for infection [11]. Poutanen et al. (2003) reported that during the 2003 SARS outbreak, 60 of the 330 SARS patients were health care workers who had direct or indirect contact with SARS-infected patients, and five of the infected healthcare workers died from SARS [7]. Even if there is a perceived high risk of infection, PCPs are willing to treat victims of emerging infectious diseases such as pandemic influenza [12] and SARS [7]. If effective infection control measures are not established, primary care providers who work in PCPs have a higher risk of contracting the infection.

The importance of pandemic preparedness for reducing the risk of infection and promoting an effective pandemic outbreak response has been highlighted in recent years. The World Health Organization (WHO) issued a “Preparedness Plan” for pandemic influenza in 1999, which was revised in 2005 [13]. The plan’s objectives and some recommendations (e.g., the use of antiviral drugs) are specific for influenza outbreaks, while other parts of the plan focus on preparedness and response to other emerging infectious diseases.

Preparedness can be defined as the ability to reduce morbidity and mortality that results from large-scale transmission of infectious diseases such as pandemic influenza, or from other natural or man-made disasters [14]. Preparedness plans consist of public health capacity building, and include activities relevant for individual healthcare facilities. These activities include surveillance, communication, vaccination services, and maintenance of an inventory of antiviral drugs. All WHO member states were advised to develop an individualized pandemic plan, because the contents and structure of healthcare partnerships depend on country-specific regulatory, finance, and administration systems [13].

Pandemic preparedness planning in Japan is based on a national preparedness plan issued by the Cabinet office and the Ministry of Health, Labour and Welfare of Japan in 2005. This plan included guidelines for pandemic influenza management, and was revised in 2009 [15] and again in 2013. The 2013 revised plan includes information on avian influenza and other novel emerging respiratory diseases such as coronaviruses. The Japanese health care system provides universal health care coverage, and patients can access any type of medical care institution, meaning that both clinics and hospitals contain PCPs. During an influenza pandemic, as a part of surveillance, every PCP is required to report a suspected pandemic influenza case to a local public health center, and take a specimen from suspected cases and transfer it to a local laboratory for PCR testing.

Pandemic preparedness plans have mainly emphasized hospital preparedness. Although there have been some attempts to include primary care, implementation of primary care preparedness and response has not been adequately addressed [16,17]. There have been few reports on the preparedness of individual PCPs to respond to emerging respiratory infectious diseases, and on the effect of planning on individual preparedness. Therefore, our aims were to: (1) examine PCP preparedness for pandemic influenza, and examine actual PCP response during the 2009 pandemic influenza outbreak, and (2) compare the preparedness of clinics versus hospitals in Japan, and evaluate the effectiveness of the current influenza plan for the promotion of institutional preparedness and response.

## Methods

We used a postal survey to assess the preparedness and response of primary care practices to the 2009 pandemic H1N1 influenza (A(H1N1)pdm09) outbreak. Survey questions were based on WHO checklists [18,19] and Japan’s guidelines for the prevention and control of pandemic influenza. The questions addressed the essential components of institutional preparedness and response to pandemic influenza [20].

The survey consisted of seven preparedness and response topics: the storage and supply of personal protective equipment (PPE), knowledge of the surveillance process, the establishment of a business continuity plan (BCP), the storage and supply of antiviral drugs, patient management to transfer severe patients to a designated hospital, the isolation of patients with influenza-like illness from other patients (such as physical barriers in shared waiting areas), and the provision of a vaccine service. The institutional information included questions related to the degree of recognition of the national preparedness plan and the degree of acceptance of A(H1N1)pdm09 patients. We included 12 questions that could be answered with yes/no or with 3- or 5-point Likert scale responses. The questionnaire was pilot-tested among a group of PCPs who were not study participants, and was updated based on the results of the pilot tests. Questionnaire details are included in Additional file 1.

The survey was conducted in Okinawa Prefecture, Japan, which has a population of approximately 1,400,000 people. We selected this prefecture as our study site because it is in close proximity to several East and Southeast Asian countries and is visited by approximately 300,000 tourists every year. As such, Okinawa Prefecture has a high risk of hosting infected patients from foreign countries and of experiencing a local epidemic of influenza or other infectious respiratory disease. Okinawa Prefecture experienced the first local epidemic of influenza A(H1N1)pdm09 in 2009, and the epidemic period of the 2009 H1N1 pandemic in Okinawa was from June 2009 to March 2010.

We identified 594 medical care institutions (clinics and hospitals) in publicly available members’ lists of the five local medical associations that cover Okinawa Prefecture. Registration with a local medical association is not compulsory for all Okinawan practices. However, only registered medical care institutions can provide government-funded influenza vaccination and join a local network to work with public health centers and local laboratories. Therefore, primary care providers seeing patients with influenza-like illness typically register with a local medical association. We used the definition of clinics and hospitals based on the definition in the Medical Care Act in Japan: a clinic is a medical care institution with 19 or fewer beds, while a hospital is a medical care institution with inpatient facilities for 20 or more patients. We defined PCPs providing care for patients with influenza-like illness as medical care institutions which contain the following departments: internal medicine, pediatrics, ear, nose and throat, and/or obstetrics/gynecology, because these are the departments where patients with influenza-like illness in Japan typically present. We made a list of all eligible PCPs from the 594 medical care institutions on the member lists, and mailed a questionnaire to all eligible PCPs (n = 465) in Okinawa Prefecture that may see patients with influenza-like illness (based on the departments listed above). The questionnaire was mailed in July 2010, shortly after the influenza A(H1N1)pdm09 pandemic. We requested that the clinic and hospital directors, who are legally required to be medical doctors in Japan, complete the questionnaire, because these administrators are responsible for pandemic influenza preparation and response. To increase the response rate, a reminder to complete the questionnaire was mailed 2 weeks after the survey was mailed. To address non-response bias, the response rate of PCPs in hospitals versus clinics was tested using chi-square analysis. Logistic regression was used to compute univariate and multivariate odds ratios (ORs) to evaluate the association between the institutional setting (hospital versus clinic) and individual aspects of pandemic preparedness, as well as recognition of the national preparedness plan. Questionnaire responses were dichotomized into ‘stocked’ and ‘not stocked’ for each PPE item, into ‘know’ and ‘not know’ for surveillance, into ‘planned’ and ‘not planned’ for BCP, into ‘implemented’ and ‘not implemented’ for isolation of symptomatic patients, into ‘provided’ and ‘not provided’ for pandemic vaccine, and into ‘stocked’ and ‘not stocked’ for antiviral drugs. Subjective evaluation of institutional preparedness was dichotomized into ‘good or fair’ and ‘not fair, bad, or not sure’. Multivariate analysis included adjustment for clinical setting (clinic versus hospital) or awareness of the national preparedness plan (yes versus no). We estimated ORs with 95% confidence intervals (95% CI), and values of P < 0.05 were considered statistically significant. Statistical analyses were performed with Stata SE software (StataCorp, College Station, TX). The institutional review board of the Tokyo Medical and Dental University approved the ethical and scientific validity of the study (ID: 1522).

## Results

### Participant characteristics

Of the 594 medical care institutions on the local medical associations’ lists, 465 institutions were eligible for enrollment in our study, and medical directors of these institutions were invited to complete the questionnaire. A total of 37.4% (174/465) responded. Eighty-six percent (149/174) of the respondents were based in clinics, and the remaining 25 were based in hospitals (Table 1). All respondents were medical doctors and 95.4% of respondents were male. The average age was 58.3 years (range: 36–90), and the average length of respondents’ experience as a physician was 31.4 years (range: 10–66). No statistically significant difference in response frequency was found between hospitals and clinics (Table 2), demonstrating that the same proportion of practices responded from each institutional setting.

**Table 1:** Characteristics of survey respondents and their institutions (N = 174)

	**Categories**	**Number**	**%**
**Respondents**			
Sex	Male	166	95.4
Age (years)	< 45	13	7.5
	45 – 64	116	66.7
	> 64	45	25.8
Length of medical experience (years)	< 20	21	12.1
	20 – 40	122	70.1
	> 40	31	17.8
Had read the government guidelines	Yes	134	77.0
Subjective evaluation of preparedness	Good or fair	95	54.6
**Institutions**			
Institutional setting	Clinic	149	85.6
	Hospital	25	14.4
Accepted patients with A(H1N1)pdm	Yes	164	94.3

N: number, A(H1N1)pdm: 2009 pandemic influenza A(H1N1).

**Table 2:** Survey response rate by institutional setting

**Institutional setting**	**Surveys sent**	**Number returned**	**Response rate (%)**
Clinics	396	149	37.6
Hospitals	69	25	36.2

Non-response in institutional setting: Pearson’s chi-square = 0.05; p = 0.823.

### Descriptive analysis

Almost all of the institutions (94.3%; n = 164) accepted patients infected with influenza A(H1N1)pdm09. More than half of the respondents (54.6%; n = 95) rated their institution’s preparedness as good or fair, regardless of their institutional setting.

Table 3 depicts the descriptive results of the seven topics included in the questionnaire. Alcohol- and non-alcohol-based hand sanitizers were well stocked (87.4%; n = 152), but recommended personal protective equipment such as N95 masks (45.4%; n = 79), gowns (30.5%; n = 53), and eye protection (21.3%; n = 37) were not adequately stocked (Table 3). Instead, PCPs stocked standard equipment such as surgical masks (84.5%; n = 147) and gloves (71.3%; n = 124) to protect themselves from infection. Anti-influenza drugs were well stocked (82.8%; n = 144). Stockpiling of antiviral drugs was a part of the medical intervention program against pandemic influenza, and these drugs were used to treat infected patients and for post-exposure prophylaxis in those who were in close contact with symptomatic individuals. In terms of surveillance, a relatively high percentage of PCPs knew the procedure for handling throat swab specimens from suspected cases, and knew that specimens should be transferred to a designated local laboratory (79.3%; n = 138). The PCPs reported that almost all of the institutions (97.7%; n = 170) have a local designated hospital for severe cases with pandemic influenza, but a relatively low percentage of PCPs (32.8%; n = 57) established a BCP for preparing for pandemic influenza.

**Table 3:** Descriptive analysis, pandemic preparedness, and response of primary care practices, Okinawa, Japan (N = 174)

	**Response**	**Number (%)**
**Healthcare staff protection**		
PPE supplies and storage		
Gown	Stocked	53 (30.46)
Mask: surgical mask	Stocked	147 (84.48)
N95 mask	Stocked	79 (45.40)
Eye protection	Stocked	37 (21.26)
Gloves	Stocked	124 (71.26)
Hand sanitizer supplies	Stocked	52 (87.36)
**Pharmaceuticals**		
Storage of antiviral drugs	Stocked	144 (82.76)
**Surveillance**		
Know how to transfer a specimen to laboratory	Knew	138 (79.31)
**Patient management**		
Know a local designated hospital for severe cases	Knew	170 (97.70)
**Institutional management**		
Business continuity plan	Planned	57 (32.76)
**Source control**		
Isolated ILI patients from other patients	Isolated	124 (71.26)
**Pharmaceutical prevention**		
Provided Vaccination service	Provided	165 (94.83)

PPE: personal protective equipment, ILI: influenza-like illness.

Many PCPs (71.3%; n = 124) isolated patients with influenza-like illness from other patients, either using different consultation rooms or a physical barrier, and almost all institutions (94.8%; n = 165) provided pandemic vaccine to their patients.

### Comparative analysis by institutional setting

After adjusting for recognition of the national pandemic preparedness plan, our multivariate regression analyses revealed that clinics were less likely to stockpile recommended high-level PPE such as N95 masks (OR 0.34; 95% CI 0.14–0.84), gowns (OR 0.15; 95% CI 0.06–0.38), and eye protection (OR 0.18; 95% CI 0.07–0.44) (Table 4). Measures for separating suspected influenza patients and the use of masks as source control against infection were adopted by fewer clinics than hospitals, but not to a significant extent.

**Table 4:** Comparison of clinic versus hospital pandemic influenza preparedness and response, Okinawa, Japan (N = 174)

		**Institutional setting**				
		**Clinic**	**Hospital**			
		**n (%)**	**n (%)**	**Univariate**	**Multivariate**	
	**Response**	**(N = 149)**	**(N = 25)**	**OR (95% CI)**	OR (95% CI)*	***p value***
**Healthcare staff protection**						
PPE supplies and storage						
Gown	Stocked	36 (24.16)	17 (68.00)	0.15 (0.06–0.38)	0.15 (0.06–0.38)	<0.00
Mask: surgical mask	Stocked	124 (83.22)	23 (92.00)	0.43 (0.10–1.94)	0.44 (0.10–1.98)	0.28
N95 mask	Stocked	62 (41.61)	17 (68.00)	0.33 (0.14–0.83)	0.34 (0.14–0.84)	0.02
Eye protection	Stocked	24 (16.11)	13 (52.00)	0.18 (0.07–0.44)	0.18 (0.07–0.44)	<0.00
Gloves	Stocked	103 (69.13)	21 (84.00)	0.43 (0.14–1.31)	0.43 (0.14–1.33)	0.14
Hand sanitizer supplies	Stocked	130 (87.25)	22 (88.00)	0.93 (0.26–3.42)	0.95 (0.26–3.50)	0.94
**Pharmaceutical arrangement**						
Storage of antiviral drugs	Stocked	120 (80.54)	24 (96.00)	0.17 (0.02–1.33)	0.17 (0.02–1.33)	0.10
**Surveillance**						
Know how to transfer						
A specimen to laboratory	Knew	121 (81.21)	17 (68.00)	2.03 (0.80–5.18)	2.42 (0.88–6.61)	0.09
**Institutional management**						
Business continuity plan	Planned	47 (31.54)	10 (40.00)	0.69 (0.29–1.65)	0.71 (0.29–1.75)	0.45
**Source control**						
Isolated ILI patients from						
Other patients	Isolated	103 (69.13)	21 (84.00)	0.43 (0.14–1.31)	0.43 (0.14–1.32)	0.14
**Pharmaceutical prevention**						
Provided vaccination service	Provided	141 (94.63)	24 (96.00)	0.73 (0.09–6.14)	0.70 (0.08–5.90)	0.74

N, n: number, CI: confidence interval, PPE: personal protective equipment, ILI: influenza-like illness.

*The multivariate odds ratio models included degree of familiarity with the national preparedness plan.

### Effect of the national preparedness plan

After controlling for institutional setting, the results of a multivariate logistic regression analysis revealed that review of the national preparedness plan was significantly associated with the establishment of a BCP (OR 5.86; 95% CI 1.97–17.45) and with knowledge about the procedure for the transfer of swab specimens to a local medical laboratory (OR 5.6; 95% CI 2.48–12.63) (Table 5). Stockpiling PPE, hand sanitizer, or antiviral drugs was not significantly associated with review of the national pandemic preparedness plan.

**Table 5:** Effect of familiarity with the national pandemic influenza preparedness plan, Okinawa, Japan (N = 174)

		**National preparedness plan**				
		**Read**	**Not read**			
		**n (%)**	**n (%)**	**Univariate**	**Multivariate**	
	**Response**	**(N = 134)**	**(N = 40)**	**OR (95% CI)**	OR (95% CI)*	***p value***
**Healthcare staff protection**						
PPE supplies and storage						
Gown	Stocked	45 (33.58)	8 (20.00)	2.02 (0.86–4.75)	2.08 (0.84–5.14)	0.11
Mask: surgical mask	Stocked	115 (85.82)	32 (80.00)	1.51 (0.61–3.78)	1.49 (0.60–3.74)	0.39
N95 mask	Stocked	65 (48.51)	14 (35.00)	1.75 (0.84–3.64)	1.74 (0.82–3.66)	0.15
Eye protection	Stocked	32 (23.88)	5 (12.50)	2.20 (0.79–6.08)	2.23 (0.77–6.44)	0.14
Gloves	Stocked	99 (73.88)	25 (62.50)	1.70 (0.80–3.58)	1.68 (0.79–3.56)	0.18
Hand sanitizer supplies	Stocked	119 (88.81)	33 (82.50)	1.68 (0.63–4.47)	1.68 (0.63–4.47)	0.30
**Pharmaceutical arrangement**						
Stockpile of antiviral drugs	Stocked	111 (82.84)	33 (82.50)	1.02 (0.40–2.60)	0.10 (0.40–2.55)	0.99
**Surveillance**						
Know how to transfer						
A specimen to laboratory	Knew	116 (86.57)	22 (55.00)	5.27 (2.38–11.70)	5.60 (2.48–12.63)	<0.00
**Institutional management**						
Business continuity plan	Planned	53 (39.55)	4 (10.00)	5.89 (1.98–17.51)	5.86 (1.97–17.45)	<0.00
**Source control**						
Isolated ILI patients from						
Other patients	Isolated	97 (72.39)	27 (67.50)	1.26 (0.59–2.71)	1.24 (0.58–2.68)	0.58
**Pharmaceutical prevention**						
Provided vaccination service	Provided	125 (93.28)	40 (100.00)	NA	NA	NA

N, n: number, CI: confidence interval, PPE: personal protective equipment, ILI: influenza-like illness, NA: not available.

*The multivariate odds ratio models included institutional setting.

## Discussion

Our results indicate that primary care practices in Okinawa Prefecture, Japan lacked essential components for pandemic influenza preparedness. Of the seven topics of pandemic preparedness and response, we found that PCPs were particularly not well prepared with respect to PPE and BCP.

PPE is recommended as the main method for protecting primary healthcare workers and patients from pandemic influenza and other infectious diseases [18,19]. However, primary care clinics were less likely than hospitals to keep adequate supplies of PPE. There was significantly less recommended high-level PPE such as N95 masks, gowns, and eye protection at clinics than at hospitals. A systematic review that investigated the efficacy of PPE for protection against respiratory viruses suggested that PPE is highly effective at preventing the spread of SARS. Odds ratios for various PPE (with a lower OR meaning more protective) are: masks (OR 0.32); N95 masks (OR 0.09); gloves (OR 0.43); gowns (OR 0.23); and hand washing, masks, gloves and gowns combined (OR 0.09) [21]. Shaw et al. [12] reported that the successful containment of SARS outbreaks depends on the appropriate use of infection control measures such as PPE [22]. In the case of influenza A(H1N1)pdm09, appropriate use of PPE, such as gloves, seemed to reduce the risk of A(H1N1)pdm09 transmission [23]. Insufficient infection control preparedness increases the probability that PCPs, especially clinic-based PCPs, will be exposed to (and contract) respiratory infectious diseases. This vulnerability can reduce the capacity of community clinics to respond to a large number of patients that require treatment during a pandemic, and puts the health of all patients attending local health care services at risk.

There are diverse reasons for the lack of PPE preparedness at primary care institutions. Clinic-based PCPs realize that PPE should be worn to protect themselves, other healthcare staff members, and patients during pandemics [12]. However, insufficient funds to purchase a large amount of PPE, and a lack of storage space, may lead to reluctance to maintain large supplies [12]. In Japan, primary care clinics are generally managed as small private practices, with limited space and small budgets. These clinics are likely to curtail the elements of preparedness that require finances and space. Most primary care providers in PCPs believe that governments are responsible for stockpiling and providing PPE during pandemics [12,24]. Without explicit guidelines that designate who is responsible for providing PPE during pandemics, supplies of PPE are likely to be inadequate. This PPE supply shortage may lead to PCPs declining to see patients with influenza-like illness, to protect themselves and their staff, which makes the personal lives and families of medical staff the priority instead of the provision of care to the community. This premise is supported by the observation that 37.5% of the general practices in Canada closed their doors during the SARS outbreak [25]. Practical incentives (e.g., financial support) and/or strategies for the distribution of stockpiled supplies of PPE should be developed by the national health authority so that individual PCPs can respond effectively during pandemics.

We found that sufficient supplies of N95 masks were not maintained by PCPs, but that adequate supplies of surgical masks were available. It is not known whether a surgical mask is as effective as an N95 mask in providing protection against influenza and other respiratory viruses. If surgical masks are sufficient, then inventories of N95 masks are not necessary. The size and nature of infectious influenza particles that are transmitted during coughing or sneezing are not well understood, and the efficacy of a surgical mask to prevent disease transmission is not known [26]. Theoretically, an N95 mask should be used for particles < 5 μm transmitted via aerosol, and the efficacy of an N95 mask for the prevention of transmission of influenza has been shown [27]. However, studies conducted in healthcare settings have not yet reached a definite conclusion regarding the use of different types of masks. During the 2009 pandemic influenza, an N95 mask was significantly associated with a lower risk of pandemic (H1N1) 2009 virus infection, compared with a surgical mask [28]. In contrast, results of a randomized trial suggested that a surgical mask does not seem to be inferior to a N95 mask for the protection of health care workers against seasonal influenza viruses [28]. The Japanese preparedness plan and guidelines do not recommend a specific type of mask, and published recommendations regarding the use of specific types of masks are not consistent [29]. The cost of N95 masks is also relevant. Phin et al. (2009) showed that N95 masks can have significant financial and logistic implications because they cost 30 times more than surgical masks and should be replaced every 3 years [30]. Considering the current lack of evidence for the superiority of N95 masks, the cost of purchasing large quantities for stockpiling, and the general lack of storage space, it seems unrealistic to prepare for pandemics with N95 masks. Overall, PPE is the most appropriate choice for limiting disease transmission, but further studies of which particular PPE is most effective are clearly necessary.

Our findings revealed that a BCP was not implemented by most clinic- or hospital-based PCPs. Institutions that reviewed the national pandemic preparedness plan and guidelines were more likely to have a BCP. A BCP is a contingency plan for responding to the surge in patient numbers during a pandemic. A BCP provides guidance on how to maintain essential functions and supplies when there are limited human resources. A BCP also identifies essential practice functions, staffing contingencies, flexible working hours and family care plans for staff members, criteria for clinic closure, ancillary staff recruiting and training, record keeping to ensure accountability for actions and inactions, and the use of antiviral medications. A BCP also includes plans for simulation exercises that complement training and that evaluate and refine local practice plans and leadership delegation [16]. Hospital BCP and clinic BCP are different, because the content of BCPs should depend on the institutional setting, size of practice, and role in the community. For instance, the US Centers for Disease Control and Prevention (CDC) [31], the American Academy of Family Physicians [32], and the Canadian Medical Association [33] issued checklists of BCP for clinics, which are relatively concise; conversely, the CDC’s checklist of BCP for hospitals is longer and more complicated [34].

It can be a major challenge to continue the essential functions of a medical practice during a pandemic. At the peak of a pandemic, approximately 30% of health care workers can be sick and absent from work [35]. In addition to the typical number of outpatients, during a pandemic a PCP may be asked to treat 50 new cases with influenza-like symptoms per week [36]. From a public health perspective, the lack of BCPs could result in disruptions in the local healthcare system, because PCPs care for approximately 90% of the common healthcare needs in a community [37,38]. Some PCPs close their clinics or hospitals during pandemics because they are not well prepared for pandemic outbreaks and they prioritize their personal life and family needs [35]. It is urgent that local health authorities should coordinate with PCPs to develop and improve clinic and hospital BCPs.

Familiarity with the national preparedness plan was positively associated with BCP establishment and with surveillance activities. BCP implementation and pandemic surveillance activities fall within the context of public health preparedness and response [16]. It is not always easy for clinicians who work on the frontline to gain practical business skills, knowledge, or an interest in the administrative functions of a medical practice. Our results suggest that improved familiarity with the national guidelines could promote public health preparedness among PCPs and lead to a systematic response to local pandemics. A previous study showed that limited awareness of the national preparedness plan is one of the major barriers to pandemic preparedness [39], and our results are consistent with this finding. One reason for the low acceptance rate of the national plan is the lack of formal PCP representation during the planning process [40]. It is critical that all primary care workers, including PCPs, community nurses, and caregivers, are engaged during preparedness plan preparation. We propose that this change could improve pandemic preparedness in the primary care sector, and PCP awareness of the plan. Furthermore, we suggest that the way in which PCPs are informed about the content and importance of the plan should be considered, to improve the number of PCPs that are aware of the national plan.

Dr. Michael Osterholm, a public health scientist and biosecurity expert, stated that “The difficulty in confronting the possibility of an H5N1 pandemic is figuring out what is necessary”. [41]. We cannot predict in advance the emergence of novel infectious diseases in a community; thus, our attention should be focused on preparation. Appropriate preparation, sufficient information, appropriate responses, administration of the correct treatment and observation when needed, and referral of patients to designated secondary hospitals can be done under the coordinated guidance of local health authorities. This type of structure is also likely to reduce the risk that an infectious disease outbreak will become a pandemic.

### Limitations

The findings of this prefecture-wide study might not represent the status of nationwide preparedness for pandemic influenza in the entire primary healthcare sector of Japan. Nevertheless, Okinawa Prefecture is known to have the highest risk in the country for local epidemics associated with emerging respiratory infectious diseases. Therefore, there is likely to be a relatively high awareness of the risks of a pandemic among PCPs in Okinawa. Thus, our findings are relevant for other prefectures and are a good estimate for the level of preparedness among PCPs in Japan.

The response rate to our survey questionnaire was relatively low (37.4%, N = 174); therefore, the results might be biased. However, our findings were similar to the findings reported by a study in the United Kingdom on the same topic [39].

Governance of the primary care sector in Japan is characterized by a loose structure that exists between clinics and hospitals. Patients with common health problems can access any PCP. Given this structure, it is not a simple matter to implement coordinated actions between PCPs and health authorities, or to optimally use PCP services [16]. This complexity may contribute to PCPs’ poor pandemic preparedness. Research is clearly needed to identify effective governance structures that facilitate individual PCP preparedness and collaborative action within the primary care sector.

We referenced scientific evidence from a variety of airborne-transmitted diseases because relatively few studies of pandemic influenza are available. In addition, there has not been universal agreement on the theoretical background of preparedness planning, and scientific evidence about which structures are most effective for implementing preparedness is still accumulating [42,43]. Nevertheless, there is agreement that preparedness in the event of a pandemic is important.

## Conclusions

The threat of pandemic influenza is persistent, and PCPs, which will be at the front line of any responses to pandemic influenza, need to properly prepare for pandemic influenza. This research provides information about pandemic preparedness and response of PCPs in Okinawa Prefecture, Japan. We assessed whether the institutional setting is associated with preparedness. PCPs, especially clinic-based PCPs, were inadequately prepared with regard to PPE. Results from this study suggest that pandemic preparedness plan awareness is likely to promote the implementation of BCPs by PCPs. Increased efforts to improve PCP familiarity with the plan, and improve PPE preparedness, are needed.

## Competing interests

The authors declare that they have no competing interests.

## Authors’ contributions

TT designed the study, collected and analyzed the data, and drafted the manuscript. YK and KK participated in the study design and provided expert advice. All authors read and approved the final manuscript.

## Pre-publication history

The pre-publication history for this paper can be accessed here:

http://www.biomedcentral.com/1471-2296/14/174/prepub

## Supplementary Material

Additional file 1 Questionnaire. Click here for file
