# Supplementary material for: Insufficient preparedness of primary care practices for pandemic influenza and the effect of a preparedness plan in Japan: a prefecture-wide cross-sectional study
Source: BMC Fam Pract. 2013 Nov 19;14:174. doi: 10.1186/1471-2296-14-174 (PMC3840630; doi:10.1186/1471-2296-14-174)
Supplement: Additional file 1 — Questionnaire. [file 1471-2296-14-174-S1.pdf]

Questionnaire on preparedness and response for pandemic influenza

Please circle the numbers that are applicable to your case.

Please answer all the questions.

We would like to ask you some questions about the "preparations" before the pandemic influenza A(H1N1) outbreak (before April 23, 2009).

1. Regarding the "preparations" for the novel influenza outbreak in your medical institution:

(Please circle only one)

1. We had prepared for the novel influenza outbreak thoroughly.
2. We had prepared for the novel influenza outbreak to some extent.
3. Not sure.
4. We had prepared inadequately for the novel influenza outbreak.
5. We had not prepared at all for the novel influenza outbreak.

2. Regarding the "Pandemic Influenza Preparedness Action Plan" (Last revised on February 17, 2009) presented by the Japanese Government:

(Please circle only one)

1. I had read it.
2. I knew about it, but did not read it.
3. I did not know about it.

3. Regarding the “Guidelines for the Prevention and Control of Pandemic Influenza” (Published on February 17, 2009) presented by the Japanese Government:

(Please circle only one)

1. I had read it.
2. I knew about it, but did not read it.
3. I did not know about it.

4. Regarding the “Pandemic Influenza Preparedness Action Plan in Okinawa” (Revised on December 2007) presented by the Okinawa Prefectural Government:

(Please circle only one)

1. I had read it.
2. I knew about it, but did not read it.
3. I did not know about it.

5. Please circle <u>all the numbers</u> indicating the “supplies” your medical institution was equipped with as part of the pandemic influenza preparedness plan:

(Please circle all items applicable to your case)

1. Gowns	2. N95 masks	3. Surgical masks
4. Goggles	5. Face shields	6. Antiseptic hand rub solution
7. Gloves	8. Anti-viral drugs	9. Other

6. Regarding the transfer of the patient samples (nasopharynx swab, throat swab, etc.) with suspected novel influenza infection to public health centers and prefectural and municipal public health institutes: (Please circle only one)

1. I knew about it.
2. I did not know about it.
3. I cannot judge.

7. Regarding a business continuity plan to alleviate situations such as excessive staff at your hospital/clinic on temporary leave of absence from duty owing to the novel influenza outbreak: (Please circle only one)

1. We planned for it.
2. We did not plan for it.
3. I cannot judge.

We would like to ask you some questions about the "response" after the pandemic influenza A(H1N1) outbreak (after April 24, 2009).

8. When did you examine the first patient with pandemic influenza A(H1N1) (including a patient with suspected pandemic influenza A(H1N1) infection)? (Please circle only one)

1. In late April 2. In early May 3. In mid-May 4. In late May

5. In early June 6. In mid-June 7. In late June 8. In early July

9. In mid-July 10. In late July 11. In early August 12. In mid-August

13. In late August 14. After September 15. No patient was examined.

9. What measures were taken for the control of health care related infections for patients with pandemic influenza A(H1N1) (including patients with suspected pandemic influenza A(H1N1) infection)?

(Please circle all items applicable to your case)

1. The waiting room for these patients was spatially differentiated from that for other patients (e.g., division of the waiting room using a physical partition and/or consulting rooms used exclusively for patients with influenza-like symptoms).
2. Additional consultation hours were set for these patients (e.g., during one hour in the afternoon, only the patients with influenza-like symptoms were examined).
3. Patients with suspected pandemic influenza A(H1N1) infection were asked to use a mask in the waiting room.
4. No special measure was taken.
5. Others

10. Regarding the use of a mask by patients with influenza-like illness in the waiting room

(Please circle only one)

1. We supplied a free mask and asked the patients to use it.
2. We supplied a mask for a fee and asked the patients to use it.
3. We did not ask the patients to use a mask.
4. Other

11. Regarding the referral institution for severely affected patients with pandemic influenza A(H1N1) infection (e.g., pneumonia, encephalitis, among others)

(Please circle only one)

1. We knew the referral institutions for severely affected patients.
2. We did not know the referral institutions for severely affected patients.
3. Our hospital was a referral institution to which severely affected patients were sent and were accepted.

12. In your medical institution, was pandemic influenza A(H1N1) vaccination conducted in outpatients?

(Please circle only one)

1. Yes
2. No
3. Not sure
